# Editorial

**DOI:** 10.1155/S1463924685000025

**Published:** 1985


Journal of Automatic Chemistry, Volume 7, Number 1 (January–March 1985), pages 1–3

### IMPORTANT ANNOUNCEMENT

**We are pleased to announce that from 1 March 1985 the *Journal of Automatic Chemistry*, published by Taylor & Francis, and *The Journal of Clinical Laboratory Automation*, previously published by Appleton-Century-Crofts, are to merge to form one journal which will be published by Taylor & Francis.**

**This is the first issue of the newly merged journal. All previous subscribers to *The Journal of Clinical Laboratory Automation* are being sent this issue, subsequent issues will be mailed on payment of their renewal invoice.**

**In connection with this merger we are pleased to announce the appointment of a Managing Editor for North America – Margaret R. Stewart, 14 Shawnee Trail, Blacksburg, Virginia 24060, USA – to whom American and Canadian articles for publication should be addressed.**

### Editorial: Computers full circle

I have written before about analytical chemistry being a great follower of fashion. The recent spate of publicity on the so-called 'LIM' computer systems (see Dr Hornstein's article below has again had me reaching for my pen to issue my usual warning to the unwary purchaser of some sophisticated computer system which purports to fulfill his every need, but may just as easily throw the laboratory and all its staff into confusion and frantic panic.

In the early 1970s I was involved in the design, development and, later, successful implementation of a laboratory computer system. Despite a great deal of resistance because of its 'central facility', it proved reliable and economic. As it evolved there came the increasing requirement to cope with instruments with built-in microprocessors and to link other computers online. Less need was in fact found for initial data reduction but also there was an increasing requirement to share, or distribute, files of data or information and add such sophisticated computing facilities as high-quality plotters.

Whilst those of us putting forward the scheme were trying to rationalize the proposal with a distributive system we were in conflict with the advocates of the 'microprocessor for everyone and everyone independent of other users' bandwagon. Needless to say, few of these people had direct experience of operating a system. What seemed to appeal to this group was that the user could be independent, whereas centralization implied dependence. In reality each of us is dependent on others and on additional information. The need to communicate is vital in all areas, particularly in laboratories. Data presented in a form an analyst accepts will have no value to a customer who wants an answer to his own questions and also some clear idea of the reliability of that data.

The distributed approach that was evolved in response to large laboratories' needs has a remarkable similarity to the various LIM systems currently being promoted. However, it does have a distinct and important difference. The instruments included within the distribution net were not specifically from one company. Also the reporting and archiving software requirements were developed specifically for individual's requirements in a particular laboratory.

Specifying the precise needs of any laboratory computer system are difficult without a clear understanding of the resources of hardware and software available. It is impossible without a detailed overview of what the laboratory requirements actually are. On the latter it is vital not to be clouded by those facilities a laboratory actually offers at present—the specification process must look beyond this aspect to the underlying requirements. A recent survey on laboratory data processing in the UK showed clearly the level of interest in implementing systems of this nature and equally clearly the lack of any real penetration at present. With increased pressure to improve productivity with reductions in manpower there will be continued pressure on management to install LIM-type systems. Given proper attention to a detailed specification the successful manager can look forward to a successful period. The unwary may invest in a very expensive and continued headache.

**Peter Stockwell**

### Comprehensive Laboratory Information Management Systems (LIMS)

*The features and advantages of LIM systems, described by Dr J. Virgil Hornstein (Assistant Product Manager, Perkin-Elmer Corporation, December 1983)*

Analytical chemistry is moving towards a goal of producing maximum analytical data with minimum sample preparation. This trend is both aided and hindered by a general decline in the number of advanced degreed analytical chemists available to industry. Perkin-Elmer recognized the emerging problem over four years ago and began the development of LIMS/2000, a laboratory information management system, which has been launched in the UK in a series of meetings. The analytical laboratory has become one of the most information intensive environments in industry. A staff of 10 to 20 can be responsible for over 50 000 samples per year. The quest for the information contained in those samples may require five to 50 independent analytical measurements. Each analytical measurement can, in turn, generate from one to as many as 100 results. The amount of data that must be reduced, correlated, and formatted for presentation is enormous. Previous developments in laboratory computerization were all focused upon ways to more rapidly produce this data, with little regard as to how to transform the data into information of use to human scientists. The first developments in laboratory information management were directed towards solving that problem. Two independent approaches had evolved: one, the automation system designed to generate more and better data; and the other, the information management system designed to transform that data into useful information. Unfortunately, the two systems were generally mutually exclusive.

Perkin-Elmer's integrated comprehensive laboratory management system was designed to provide the user with the capability of collecting and reducing data, collating results, and producing reports. A portion of the software is designed to follow, or track, the sample as it moves physically and analytically through the laboratory. A query package is provided to allow the user to rapidly determine the status of a particular sample and the results of the analyses assigned to it. Reports can be generated and directed to any output device on the system. LIMS/2000 provides an archival storage function and a retrieval function that allows the user to store historical records on bulk media, such as magnetic tape, and restore them to the system whenever necessary. In heavily regulated industries, such as pharmaceutical manufacturing, the ability to store and retrieve data is required by federal regulation. Of course not all laboratories are so highly automated that there are no manual procedures so a portion of the LIMS/2000 software was designed to provide for manual entry of analytical data. User-defined prompts, specific to each procedure, guide the analyst through the data-entry task. Software linkages to user-written programs for further processing of the data are also provided.

To be a *comprehensive* laboratory management system, LIMS/2000 must also provide easy means to enter analytical data. The manual entry of data was discussed earlier but most labs have utilized automated instrumentation. The entry of several thousand data points for each analytical procedure is unacceptable. LIMS/2000 provides two paths for the capture and processing of data from analytical instruments. One path, represented by C/LAS (Chromatography Laboratory Automation System), utilizes a centralized processing scheme. Data are collected from each chromatograph, digitized, and transmitted to the LIMS/2000 processor. C/LAS then processes the data and reports the results, as well as updating all entries within LIMS/2000 that are affected by the process. The second path, represented by our LIMSCOMM package, allows the analytical data processing to be performed by the local work-station, either a 3600 or Sigma 15 Data Station, or a Series 7000 Professional Computer, using one of the over 40 analytical application packages developed by Perkin-Elmer. This approach is known as distributed processing. Perkin-Elmer chose to offer both paths on LIMS/2000 because the typical analytical laboratory *requires* some mix of centralized and distributed processing. When data or results are transmitted to LIMS/2000 via LIMSCOMM, further processing can be performed. LIMS/2000 will also check all other entries affected by the procedure and update them. The user can also recall data from LIMS/2000 for reanalysis on any data station (supporting the particular analytical software package) in the laboratory, not just the original one.

LIMS/2000 is able to function in all these roles—data manager, data processor, reports generator, etc.—because it is built upon a data-base management package. All data is stored in a central location, the LIMS/2000 data-base, and is available to all parts of LIMS/2000. The use of a data-base management system (DBMS) gives LIMS/2000 data independence: all applications 'see' the data as the application wishes to see it. Before DBMS technology evolved, companies wrote programs specific to each application. Data were formatted in various application-dependent forms. Interchange of information among programs became a very difficult process: what is the item called, where is it, how is it formatted, how must it be accessed, and so on. LIMS/2000, with its use of a DBMS, avoids all these problems. This allows Perkin-Elmer to add and/or customize applications to meet the general needs of the market or the specific requirements of a single user.

The discussion has focused upon one type of information critical to laboratory operation: scientific data. There is another type of information that has, in recent years, become equally important. That is the financial and operational information. A laboratory manager must be able to measure how well the laboratory is operating. LIMS/2000 is developing a Laboratory Resource Planning (LRP) package to provide these answers. LRP will measure cost per analysis, sample turnaround time, time-charge billing, equipment utilization, and work-load. This information, while not critical to the users of the laboratory's services, is crucial to the operation and management of the laboratory. When all factors are considered, the benefits of installing a comprehensive laboratory management system, such as LIMS/2000, are substantial. A few of the more obvious benefits are:

(1) Automatic sample numbering
(2) Standardized sample log-in menus
(3) Verification of information entered against a list of valid entries
(4) Automatic test assignment for routine samples
(5) Automatic generation of a receipt for the submitter
(6) Automatic production of labels
(7) Generation of a report on sample distribution
(8) Generation of work-lists with samples in the order defined by the user
(9) Generation of work orders
(10) Automated data collection and reduction
(11) Automatic comparison of results with an acceptable range
(12) Prompting for manual data entry
(13) Electronic filing of standard tests and methods
(14) Easy inspection of previously collected data
(15) Easy time charge entry
(16) Backlog reports for scheduling purposes
(17) Exception reports
(18) Easy inspection of results for supervisor's approval
(19) Sample and test status checks
(20) Sample location tracking
(21) Rapid response to submitter inquiries
(22) Reports on performance, by department:
    Turnaround time
    Productivity
    Quality
(23) Non-routine searches for information
(24) Automatic report generation
(25) Word processing
(26) Long-term filing of data
(27) Easy graphical display and manipulation
(28) On-line programming capability
(29) Inspection of work profiles by customer, test, or sample type
(30) Work-load projections
(31) Cost allocations
(32) Automatic billing
(33) Reduction in paperwork
(34) Reduction in errors
(35) Increased ability to answer inquiries from higher management.

A review of the list shows that a comprehensive system provides benefits in all areas of a laboratory's operation. The familiar task of data collection and reduction is only one small part of the comprehensive LIMS package.

LIMS/2000 offers other benefits: the decision to base LIMS/2000 upon a DBMS means that Perkin-Elmer can continue to add new applications and features without making the user's existing system or data obsolete. The concurrent support of both centralized and distributed processing gives the user ultimate flexibility in system design and construction. LIMS/2000 was designed for easy customization, so that it can be modified to meet each laboratory's particular requirements.

*Information from (UK) Perkin-Elmer Ltd, Post Office Lane, Beaconsfield, Buckinghamshire HP9 1QA, tel.: 04946 6161; (USA) Perkin-Elmer Corporation, Analytical Instruments, Main Avenue (MS-12), Norwalk, Connecticut 06856, tel.: 203 762 1000; (FR Germany) Bodenseewerk Perkin-Elmer & Co., GmbH, Postfach 1120, 7770 Ueberlingen, tel.: 07551 811.*

### FLOW ANALYSIS III

The Third International Conference on Flow Analysis will be held in Birmingham, UK from 5 to 8 September 1985, organized by the Midlands Region of the Analytical Division of the Royal Society of Chemistry. The scope of the conference will be similar to the previous Flow Analysis Meetings (in Amsterdam in 1979 and in Lund in 1982) and current research on all aspects of continuous flow analysis will be covered, including:

Instrumentation for flow injection analysis and for continuous segmented and unsegmented flow analysis, and approaches to total automation; New detector systems and hybrid systems; Theory of flow analysis; Applications in industrial, environmental and clinical analysis.

Invited lecturers include Dr Stewart, Professors Riley and Ružička. Papers for inclusion can be submitted up to 15 April to Dr A. M. G. Macdonald, Department of Chemistry, The University, PO Box 363, Birmingham B15 2TT, UK.

*Registration forms are available now from The Secretary, Analytical Division, Royal Society of Chemistry, Burlington House, London W1V 0BN.*

